# Impact of Prosthetic Material and Restoration Type on Peri-Implant Bone Resorption: A Retrospective Analysis in a Romanian Sample

**DOI:** 10.3390/jcm13061794

**Published:** 2024-03-20

**Authors:** Denisa Tabita Sabău, Raluca Iulia Juncar, Abel Emanuel Moca, Teofana Bota, Rahela Tabita Moca, Mihai Juncar

**Affiliations:** 1 Doctoral School of Biomedical Sciences, University of Oradea, 1 Universității Street, 410087 Oradea, Romania; denisa.sabau@gmail.com (D.T.S.); teofana.bota@gmail.com (T.B.); rahelamoca@gmail.com (R.T.M.); 2 Department of Dentistry, Faculty of Medicine and Pharmacy, University of Oradea, 10 Piața 1 Decembrie Street, 410073 Oradea, Romania; mihaijuncar@gmail.com

**Keywords:** peri-implant bone resorption, metal-ceramic, zirconia, Romanian population

## Abstract

**Background/Objectives:** This research investigates the nuanced factors influencing peri-implant bone resorption in implant-supported fixed prostheses, with a focus on age, gender, implant location, time since prosthetic loading, and material characteristics. **Methods:** Records from a dental clinic in Oradea, Romania, between 1 January 2017 and 1 January 2023, were scrutinized and were selected by means of purposive sampling. All records were analyzed between 1 May 2023 and 15 June 2023. A total of 160 implants were included, and the prosthetic restorations were either metal-ceramic or zirconia. Implants from a single manufacturer were used, and a standardized loading protocol was followed. The study examined variables such as age, gender, implant location, prosthetic material, and time since prosthetic loading. **Results:** A total of 160 implants were included, with 78 applied to female patients (48.8%) and 82 to male patients (51.2%). The age range of the patients undergoing dental implant procedures was 30 to 79 years. Implants were distributed between the mandible (51.2%) and maxilla (48.8%), with 49.4% placed in the posterior dental arches and 50.6% in the anterior dental arches. The majority of patients received metal-ceramic prosthetic reconstructions (76.9%). Statistical analysis revealed significant differences in resorption patterns between zirconia and metal-ceramic restorations (*p* < 0.001), with zirconia restorations exhibiting higher resorption in the mesial-vertical and distal-vertical planes compared to metal-ceramic restorations. Age-related factors showed a significant association with distal-vertical resorption (*p* = 0.017), with patients aged 60–69 years exhibiting higher resorption values compared to those aged 40–49 years. Gender differences were observed in mesial-horizontal resorption (*p* = 0.036), with male patients displaying higher resorption values compared to female patients. Implant location and time elapsed since implant loading did not show significant associations with resorption patterns. **Conclusions:** The study provides insights into the multifactorial nature of peri-implant resorption. Age, gender, and material characteristics contribute to variations, informing personalized treatment approaches. The findings facilitate a comprehensive understanding for clinicians, enhancing treatment planning and post-operative care.

## 1. Introduction

Dental prosthetics, defined in 1998 by Jokstad et al. as the discipline of dentistry concerned with the consequences of congenital absence or acquired loss of oral tissues and with the methods for assessing whether more benefit than harm is derived from inserting artificial devices made from alloplastic materials [1], is an ever-evolving field. Modern materials and technologies within dental prosthetics now offer enhanced resistance, improved aesthetics, and heightened comfort for both practitioners and patients [2]. Treatment options vary based on factors such as the number, prognosis, and location of remaining teeth, as well as patient expectations and financial considerations [3].

The advent of dental implantology has significantly benefited patients in need of dental prostheses, allowing for applications ranging from single-tooth replacements to complex cases involving complete edentulism [4]. Despite potential higher costs in complex cases compared to conventional prosthetic options like complete dentures [5], the long-term advantages of dental implants are substantial. One of the positive aspects of dental implants is their conservative nature. Dental implants replace prosthetics on natural teeth, thereby avoiding the sacrifice of hard dental tissue. Unlike natural tooth abutments that are prosthetically restored and can develop complications such as secondary caries, necrosis, pulpitis, acute or chronic apical periodontitis, dental implants eliminate these risks [6]. Supra-implant prosthetic rehabilitation offers advantages in improving patients’ self-perception and masticatory efficiency. Tooth loss and both mobile and fixed prosthetic rehabilitation negatively impact patients’ quality of life. They report decreased social confidence, disturbed self-image, lowered self-esteem, and reduced masticatory efficiency. However, these aspects are improved or eliminated with prosthetic rehabilitation on implants [7]. In edentulous patients, dental implants provide the advantage of a more stable and retentive prosthetic reconstruction, particularly in the mandibular arch [8]. Improved aesthetics and speech are additional advantages of implant-supported prosthetic rehabilitation [9]. All of these advantages contribute to an improved oral health-related quality of life [10]. Additionally, dental implants typically boast a high long-term success rate, exceeding 90% [11]. Conventional prosthetics rely on the utilization of remaining teeth for patients requiring fixed methods of prosthetic rehabilitation. These teeth can undergo endodontic treatment, with a success rate of approximately 80% [12]. However, in cases of teeth afflicted with periodontal disease, the success rate may be even lower, as indicated by studies [12]. All these aspects led to a preference for implant-supported prosthetics among both dentists [13] and patients [14].

In implant-supported fixed prosthetics, dental implants can support individual dental crowns in cases of single-tooth absence [15] or contribute to the construction of fixed-full arch prostheses [16]. The materials used for designing implant-supported restorations are continually evolving. While conventional materials such as monomethacrylate or acrylic resins, methacrylates, or bis-acryl/composite resins, as well as modern materials like milled PMMA (poly (methyl methacrylate)) and 3D-printed resins, are used for interim implant-supported prostheses, the most commonly utilized materials for definitive implant-supported prostheses are metal-ceramic and zirconia [17]. Different abutment materials have been used in order to determine their effect on the peri-implant inflammation [18,19], but histological analysis of biopsies from patients with zirconia and titanium abutments revealed no distinct differences with respect to peri-implant soft tissue health, and it was concluded that the perceived differences in appearance of soft tissue overlaying the abutments could be due to optical properties rather than biological differences [20]. Moreover, the mechanical properties of the abutment material seem to be more important for the survival of implant reconstruction rather than their esthetic outcome [19]. Certain authors recommend the use of zirconia abutment in the anterior sector, especially in cases where, due to lack of bone, the implant has been positioned with a buccal tilt. In these cases, we can choose to prioritize the aesthetics due to the high visibility of the reconstruction. An anatomically thin gingiva at the cervical level, in some cases, can make the material positioned under the cervical margin visible. Zirconia is a white material, so it masks better than titanium under the upper limit of the gingival margin, and also provides high strength values for anterior sector. The use of this type of abutment in the posterior sector would be extremely risky given the mechanical requirements in this area [21]. 

Metal-ceramic restorations serve as a viable option for supra-implant prosthetic rehabilitation. However, they are susceptible to chipping and ceramic fracture, attributed to impact and fatigue loads, occlusal forces, or improper design, among other factors. In contrast, zirconia restorations exhibit superior strength, toughness, and aesthetics compared to metal-ceramic counterparts. Additionally, they entail a reduced presence of metal in the oral cavity [22]. Despite both variants being viable treatment options [23], zirconia, a biocompatible material with high flexural strength [24], is increasingly employed in the construction of implant-supported fixed prostheses, with ceramic-veneered zirconia restorations offering superior aesthetics compared to metal-ceramic prostheses [17]. The integration of CAD/CAM technology in dentistry has further facilitated the development of zirconia-based implant-supported fixed prostheses [17].

Despite the numerous advantages of implant-supported fixed prosthodontics, implant failure remains a concern in certain cases [24]. Etiological factors such as infection, impaired healing, and overload contribute to implant failure [24]. Peri-implantitis, an inflammation of the peri-implant mucosa, poses a significant risk [25]. Marginal bone loss, while considered essential in peri-implantitis development, does not invariably lead to this condition [25]. Factors such as physiological remodeling after surgery or prosthetic loading, poor implant placement, implant design, and complex surgical techniques can contribute to marginal bone loss [25]. Emerging from the literature the platform-switching concept is a crucial factor that lessens the marginal bone level, and the biological width contributes to the maintenance of hard and soft tissues. The implant–abutment junction is positioned internally thus it helps marginal bone preservation, internal positioning makes inflammatory invasion distant from the bone and forms a plane of biological width. Moreover, this lessens bone-level reduction by altering the micro-space from the marginal bone [26]. Bone remodeling appears to depend on a particular infectious area of the soft tissue surrounding the dental implant [27].

The interface between the abutment and the implant is, lately, highly studied in the literature. A lot of knowledge has surfaced as a result. While using platform switching the placement of the implant abutment junction is more stable and fit [28]. Implants with an internal tapered connection have demonstrated clinically less marginal bone remodeling during the first and consecutive years post-surgery [28].

The advent of dental implantology has revolutionized prosthetic treatment, offering solutions from single-tooth replacements to complex full-arch restorations. Despite their long-term advantages, including improved stability and aesthetics, challenges such as implant failure and peri-implant complications persist. While peri-implantitis and bone resorption have been extensively studied, few investigations have explored the potential influence of the material used in implant-supported fixed prostheses on peri-implant resorption. Additionally, limited studies have examined the impact of prosthetic restoration type on peri-implant bone resorption, with these studies being scarce in the Romanian population.

Hence, the aim of this retrospective study was to comprehensively investigate the clinical outcomes and bone resorption patterns associated with dental implant procedures and subsequent prosthetic reconstructions over a period of 5 years, in a sample of patients from Romania. The study sought to elucidate the factors influencing the success rates and longevity of implant supported prosthetic reconstructions, as well as to evaluate the impact of various demographic and clinical variables on treatment outcomes, and in this way to provide valuable insights into the clinical outcomes and factors influencing the success of dental implant procedures and prosthetic reconstructions, ultimately contributing to the optimization of patient care and treatment protocols in dental implantology and prosthodontics.

## 2. Materials and Methods

### 2.1. Ethical Considerations

The study obtained approval from the Ethics Committee of the University of Oradea (IRB No. CEFMF/1 from 13 April 2023) and adhered to the guidelines set forth in the 2008 Helsinki Declaration and its subsequent amendments.

### 2.2. Sample Selection

This retrospective study involved a thorough examination of medical records belonging to patients who underwent dental implant procedures and subsequent prosthetic reconstructions. Records from a dental clinic in Oradea, Romania, between 1 January 2017 and 1 January 2023, were scrutinized and were selected by means of purposive sampling. All record were analyzed between 1 May 2023 and 15 June 2023.

Inclusion criteria encompassed patients aged 18 or older, with at least one implant utilized for prosthetic restoration during the stipulated period. Only individuals who provided informed consent for the anonymous use of their data for scientific purposes were included. 

Exclusion criteria comprised patients who failed to attend regular check-ups every six months, those receiving implants or prosthetic reconstructions from other dental clinics, and those lacking crucial data in their medical records concerning prosthetic works or implants. Patients with missing data necessary for the study’s development were also excluded. Subsequently, patients with implants placed by oro-maxillofacial surgeons other than M.J. and those whose prosthetic reconstructions were completed by dental prosthetics specialists other than R.I.J. and D.T.S. were excluded. 

All utilized implants were Implant Swiss (Novodent SA, Yverdon-les-Bains, Switzerland), with surfaces etched using the Double Acid Etching technique to enhance osseointegration. These are implant with an internal tapered connection and platform switching. Implants considered to have a positive evolution at six months exhibited stability during clinical examination, lacked mobility, and showed no signs of pain, infection, or symptoms indicating nerve damage as reported by patients. Prosthetic loading adhered to a three-month post-insertion loading time across all cases.

Variables considered in the analysis included gender (male, female), age groups (30–39 years, 40–49 years, 50–59 years, 60–69 years, 70–79 years), implant location (maxilla, mandible), position on the dental arch (anterior area, posterior area), prosthetic reconstruction material (metal-ceramic, zirconia), type of prosthetic restoration (single crown, connected crowns, bridge), and time elapsed since completion of the prosthetic reconstruction (0.5–1 year, 1–2 years, 2–3 years, 3–4 years, 4–5 years, more than 5 years). To mitigate bias, two investigators independently reviewed all medical records.

### 2.3. Assessment of Bone Resorption

Before implant placement, patients underwent a 3D Cone Beam Computed Tomography (CBCT) evaluation. Subsequent radiological assessments were conducted immediately post-implant placement and again at the 6-month mark. Following implant loading, CBCT evaluations were performed at a 6-month interval. Bone resorption measurements were conducted on calibrated CBCT radiographs obtained at various stages post-completion of prosthetic work. Both vertical and horizontal measurements were taken on the mesial and distal surfaces of the implant. The implant-abutment interface served as a reference line, with distances expressed in millimeters measured to the first point of bone contact with the implant. 

### 2.4. Statistical Analysis

Statistical analyses were conducted using IBM SPSS Statistics 25 and Microsoft Office Excel/Word 2013. Quantitative variables were assessed for distribution using the Shapiro-Wilk test and were presented as means with standard deviations or medians with interpercentile ranges. Non-parametrically distributed quantitative independent variables were analyzed using the Mann-Whitney U/Kruskal-Wallis H test, supplemented by Dunn-Bonferroni post hoc tests. The Friedman test, accompanied by post hoc Dunn-Bonferroni tests, was employed for non-parametrically distributed quantitative variables with repeated measures. Repeated measures ANOVA, augmented by Bonferroni post hoc tests, was used for quantitatively repeated measures with normal distribution. Statistical significance was set at a *p*-value of <0.05.

## 3. Results

### 3.1. Sample Characteristics

**Figure 1:**
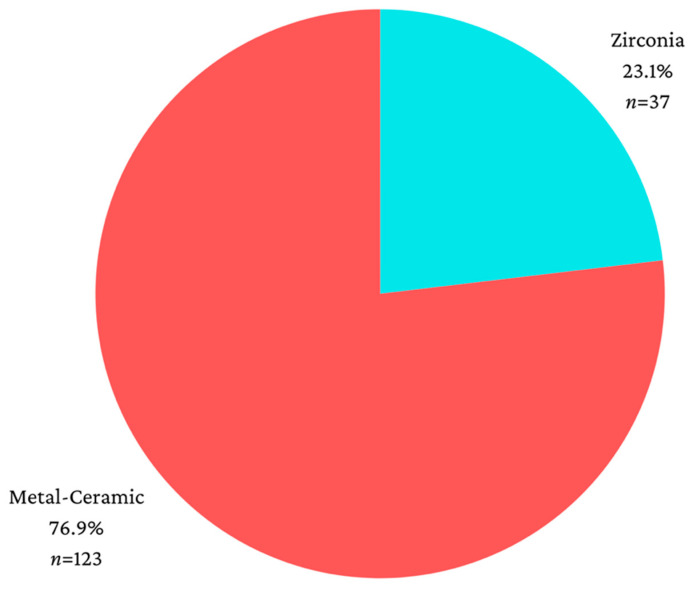
Distribution according to the type of material used for restoration.

The study included 160 implants, with 78 applied to female patients (48.8%) and 82 applied to male patients (51.2%). The patients undergoing dental implant procedures ranged in age from 30 to 79 years. Regarding implant location, 82 implants (51.2%) were in the mandible, while 78 (48.8%) were in the maxilla. Additionally, 79 dental implants (49.4%) were placed in the posterior dental arches, and 81 implants (50.6%) were in the anterior dental arches. The majority of patients received metal-ceramic prosthetic reconstructions (76.9%) (Figure 1).

### 3.2. Material Used for Prosthetic Restoration and Amount of Resorption

The data in Table 1 represent the comparison of resorption in different planes related to the type of material used for long-term prosthetic reconstruction. For the mesial-vertical plane, the distribution of resorption was non-parametric in all groups according to the Shapiro-Wilk test (*p* < 0.05), but the differences between groups were not significant according to the Mann-Whitney U test (*p* = 0.355). Therefore, resorption in the mesial-vertical plane was not significantly different compared to the reconstruction material.

For the distal-vertical plane, the distribution of resorption was non-parametric in the group of implant-supported metal-ceramic restorations according to the Shapiro-Wilk test (*p* < 0.001). The differences between groups were not significant according to the Mann-Whitney U test (*p* = 0.456), indicating that resorption in the distal-vertical plane was not significantly different compared to the reconstruction material.

For the mesial-horizontal and distal-horizontal planes, the resorption distribution was non-parametric in all groups according to the Shapiro-Wilk test (*p* < 0.05). The differences between the groups were not significant according to the Mann-Whitney U test (mesial-horizontal—*p* = 0.794; distal-horizontal—*p* = 0.932). Therefore, mesial and distal resorption in the horizontal plane were not significantly different compared to the reconstruction material.

The data in Table 2 represent the comparison of resorption values from the mesial/distal level in the horizontal/vertical planes in implant-supported zirconia or metal-ceramic prosthetic restorations. The distribution of resorption was non-parametric in most measurements according to the Shapiro-Wilk tests (*p* < 0.05). According to the Friedman test, the differences between the resorption measurements were significantly different (*p* < 0.001), and the post hoc tests showed the following:

- For zirconia restorations, resorption in the mesial-vertical plane (median = 1.65, IQR = 0.93–2.43) was significantly higher than resorption in the mesial-horizontal plane (median = 0.56, IQR = 0–1.10) (*p* < 0.001) or compared to the resorption in the distal-horizontal plane (median = 0.51, IQR = 0–1.07) (*p* = 0.001).
- For zirconia restorations, resorption in the distal-vertical plane (median = 1.76, IQR = 1.01–2.57) was significantly higher compared to resorption in the mesial-horizontal plane (*p* < 0.001) or compared to the resorption in the distal-horizontal plane (*p* < 0.001).
- For metal-ceramic restorations, resorption in the mesial-vertical plane (median = 1.36, IQR = 0.24–2.35) was significantly higher than resorption in the mesial-horizontal plane (median = 0.54, IQR = 0–0.91) (*p* < 0.001) or compared to the resorption in the distal-horizontal plane (median = 0.49, IQR = 0–1.01) (*p* < 0.001).
- For metal-ceramic restorations, resorption in the distal-vertical plane (median = 1.65, IQR = 0.54–2.47) was significantly higher compared to the resorption in the mesial-horizontal plane (*p* < 0.001) or compared to the resorption in the distal-horizontal plane (*p* < 0.001).

**Table 2:** Mesial/distal—horizontal/vertical resorption for zirconia or metal-ceramic restorations.

	Resorption	Mean ± SD	Median (IQR)	*p* *
**Zirconia**	Mesial-Vertical (*p* = 0.007 **)	1.83 ± 1.42	1.65 (0.93–2.43)	<0.001
	Distal-Vertical (*p* = 0.466 **)	1.74 ± 1.03	1.76 (1.01–2.57)	
	Mesial-Horizontal (*p* = 0.002 **)	0.64 ± 0.62	0.56 (0–1.10)	
	Distal-Horizontal (*p* < 0.001 **)	0.61 ± 0.63	0.51 (0–1.07)	
**Metal-Ceramic**	Mesial-Vertical (*p* < 0.001 **)	1.62 ± 1.49	1.36 (0.24–2.35)	<0.001
	Distal-Vertical (*p* < 0.001 **)	1.73 ± 1.51	1.65 (0.54–2.47)	
	Mesial-Horizontal (*p* < 0.001 **)	0.65 ± 0.77	0.54 (0–0.91)	
	Distal-Horizontal (*p* < 0.001 **)	0.62 ± 0.69	0.49 (0–1.01)	

SD—Standard deviation, IQR—interquartile range, * Related-Samples Friedman’s Two-Way Analysis of Variance by Ranks, ** Shapiro-Wilk Test, significance level at *p* < 0.05.

### 3.3. Type of Prosthetic Restoration and Amount of Resorption

For resorption in the mesial-vertical plane (*p* = 0.217), mesial-horizontal plane (*p* = 0.055), and distal-horizontal plane (*p* = 0.283), the differences between the groups were not significant. Therefore, the resorption in these planes was not significantly different related to the type of prosthetic restoration (Table 3).

In the case of distal-vertical plane resorption, the distribution was non-parametric according to the Shapiro-Wilk test (*p* < 0.05), and the differences between groups were significant according to the Kruskal-Wallis H test (*p* = 0.026). Post hoc tests revealed that implant-supported dental bridges had significantly higher resorption values compared to implant-supported connected crowns (Table 3).

Regarding the comparison of resorption values from the mesial/distal level in the horizontal/vertical plane in implant-supported single crowns, implant-supported connected crowns, or implant-supported dental bridges, it was observed that the distribution of resorption was non-parametric in most measurements according to Shapiro-Wilk tests (*p* < 0.05). According to the Friedman test, the differences between the resorption measurements were significantly different (*p* < 0.001), and the post hoc tests showed the following:

- For single crowns, the resorption from the mesial-vertical plane (median = 1.58, IQR = 0.24–2.31) was significantly higher than the resorption from the mesial-horizontal plane (median = 0.50, IQR = 0–0.95) (*p* < 0.001) or compared to the resorption from the distal-horizontal plane (median = 0.56, IQR = 0–1.19) (*p* = 0.001). Additionally, resorption from the distal-vertical plane (median = 1.74, IQR = 0.99–2.47) was significantly higher compared to the resorption from the mesial-horizontal plane (*p* < 0.001) or compared to the resorption from the distal-horizontal plane (*p* < 0.001).
- For connected crowns, the resorption from the mesial-vertical plane (median = 1.22, IQR = 0–2.34) was significantly higher than the resorption from the mesial-horizontal plane (median = 0.53, IQR = 0–0.94) (*p* < 0.001) or compared to the resorption from the distal-horizontal plane (median = 0.31, IQR = 0–1.02) (*p* < 0.001). Furthermore, resorption from the distal-vertical plane (median = 1.39, IQR = 0–1.99) was significantly higher compared to the resorption from the mesial-horizontal plane (*p* < 0.001) or compared to the resorption from the distal-horizontal plane (*p* < 0.001).
- For dental bridges, the resorption from the mesial-vertical plane (median = 1.86, IQR = 0.74–2.79) was significantly higher than the resorption from the mesial-horizontal plane (median = 0.68, IQR = 0.42–1.10) (*p* = 0.007) or compared to the resorption from the distal-horizontal plane (median = 0.39, IQR = 0.22–0.87) (*p* < 0.001). Moreover, resorption from the distal-vertical plane (median = 1.90, IQR = 0.80–3.03) was significantly higher compared to the resorption from the mesial-horizontal plane (*p* < 0.001) or compared to the resorption from the distal-horizontal plane (*p* < 0.001) (Table 4).

### 3.4. Influence of Age, Gender, Implant Location, and Time Elapsed since Prosthetic Loading on Resorption

The analysis of age-related factors revealed no significant differences in resorption in the mesial-vertical (*p* = 0.060), mesial-horizontal (*p* = 0.491), or distal-horizontal (*p* = 0.446) planes. However, a notable finding emerged for resorption in the distal-vertical plane (*p* = 0.017). Patients aged between 60–69 years exhibited significantly higher resorption values compared to those aged between 40–49 years. Specifically, patients aged 40–49 years displayed an average resorption value of 1.29 mm (median = 1.24, IQR = 0.3–1.91), while patients in the 60–69 age group had a mean resorption value of 2.21 mm (median = 2, IQR = 1.51–2.7).

Gender did not significantly impact resorption in the mesial-vertical (*p* = 0.163), distal-vertical (*p* = 0.186), or distal-horizontal (*p* = 0.157) planes. However, a notable difference emerged in the mesial-horizontal plane (*p* = 0.036). Male patients displayed higher mesial-horizontal resorption values compared to female patients. Specifically, female patients had an average mesial-horizontal resorption of 0.52 mm (median = 0.49 mm, IQR = 0–0.80), while male patients had an average mesial-horizontal resorption of 0.78 mm (median = 0.64, IQR = 0–1.13).

The location of the implant did not significantly influence resorption in any plane, whether the implants were in the maxilla or mandible, or in the anterior or posterior area. In all these instances, the *p*-value exceeded 0.05.

The time elapsed since implant loading did not significantly influence resorption in the mesial-horizontal (*p* = 0.621), distal-horizontal (*p* = 0.965), mesial-vertical (*p* = 0.536), or distal-vertical (*p* = 0.427) planes.

## 4. Discussion

The present study aimed to assess various factors influencing resorption patterns around dental implants and their prosthetic reconstructions. Our investigation involved 160 implants, distributed across different patient demographics and implant characteristics. The patient sample encompassed a balanced representation of gender spanning an age range of 30 to 79 years. Analysis of resorption in different planes concerning the type of prosthetic material revealed intriguing insights. While no significant differences were observed in resorption across various materials in the mesial-vertical, distal-vertical, mesial-horizontal, and distal-horizontal planes, distinctive trends emerged concerning zirconia and metal-ceramic restorations. Zirconia restorations exhibited significantly higher resorption in the vertical planes compared to horizontal planes, whereas metal-ceramic restorations displayed a similar pattern. Further investigation into different prosthetic designs elucidated nuanced differences in resorption patterns. Notably, implant-supported dental bridges demonstrated significantly higher resorption in the distal-vertical plane compared to connected crowns. Additionally, single crowns, connected crowns, and dental bridges exhibited varying degrees of resorption across different planes, suggesting the importance of prosthetic design in influencing peri-implant bone changes. The present study contributes to the understanding of peri-implant resorption dynamics in implant-supported fixed prostheses, shedding light on the influence of age, gender, implant location, and time elapsed since prosthetic loading. These findings offer critical insights into the multifactorial nature of long-term outcomes in implant-supported prosthetic reconstructions.

Our analysis indicates a noteworthy association between age and peri-implant resorption, particularly in the distal-vertical plane. Patients in the 60–69 age group exhibited significantly higher resorption values compared to their 40–49 counterparts. This observation aligns with existing literature highlighting age as a relevant factor in predicting and managing peri-implant complications. Numerous studies have underscored a correlation between advanced age and the occurrence of peri-implantitis [29,30]. Several factors, more prevalent among older adults, contribute to this association, including challenges in maintaining optimal oral hygiene [31], diminished masticatory function [32], and a decline in bone mineral density [33]. Age emerges as a pivotal determinant in bone health, given the well-established trend of decreasing bone mass density with aging, observed in both male and female patients. Moreover, age-related bone loss predominantly affects the cancelous compartment. The underlying mechanisms, including heightened oxidative stress, exert direct control over osteoclast activity on trabecular bone. It’s noteworthy that these mechanisms have a more limited impact on the cortical bone [34]. This nuanced understanding of age-related bone dynamics is crucial in comprehending the multifaceted nature of peri-implant complications and underscores the importance of tailored interventions for older individuals undergoing implant-based dental procedures. The association between age and distal-vertical resorption, as well as gender differences in mesial-horizontal resorption, suggests the need for personalized treatment approaches based on patient demographics. Older patients may require closer monitoring for peri-implant bone changes, particularly in the distal-vertical plane.

While gender did not significantly impact resorption in most planes, a significant difference emerged in the mesial-horizontal plane. Male patients displayed higher mesial-horizontal resorption values compared to female patients. This gender-related variance prompts further investigation into potential anatomical or biological factors influencing implant-supported fixed prosthesis outcomes in male and female patients. The observed outcomes can be partially elucidated by the tendency of male patients to be less proactive in seeking preventive dental care and more prone to neglecting their oral health. Furthermore, men tend to have fewer regular dental visits compared to women, often consulting a dentist primarily for acute issues [35]. Notably, the literature search did not reveal other studies specifically examining age or gender as distinct factors influencing bone resorption in the specified planes (mesial-vertical, distal-vertical, mesial-horizontal, distal-horizontal). This gap highlights the novelty of our investigation in considering these parameters within the context of peri-implant outcomes.

Surprisingly, the location of the implant did not significantly influence resorption in any plane, whether in the maxilla or mandible or in the anterior or posterior area. These results challenge conventional assumptions and underscore the need for a more nuanced understanding of individual patient characteristics that may contribute to peri-implant resorption. Clinicians should reassess traditional paradigms regarding implant success criteria and consider other factors such as occlusal forces, oral hygiene, and systemic health status when evaluating peri-implant bone health over time. Revealing evidence has proven that the tissues surrounding implants mount a greater host-inflammatory response to plaque accumulation than teeth [28]. Coronal cervical margin placement plays an important role in the evolution of peri-implant tissues. When the cervical margin is placed at the same level as the coronal level of the peri-implant sulcus the biofilm can very easily be removed from the proximity supporting tissue health [28]. Similarly, a study conducted in 2015 found no significant difference in crestal bone loss between maxillary and mandibular implants across various sites after one year of functional loading. However, it did reveal higher crestal bone loss for anterior implants compared to posterior ones [36]. In a comprehensive evaluation of short dental implants placed in partially edentulous patients over a 15-year period, Anitua and Alkhraisat (2019) reported significantly greater bone loss in the maxilla [37]. Additionally, Vidal et al. identified a higher incidence of implant failure in the upper arch [38]. Nevertheless, it is noteworthy that studies indicating increased bone loss in the lower arch have also been documented [39].

In contrast to some expectations, the time elapsed since prosthetic loading did not emerge as a significant factor influencing resorption. This finding is reassuring for clinicians and patients, suggesting that the duration between prosthetic loading and subsequent resorption may not be a critical determinant. The original flat to flat external connection was reported to allow wider micromovements and also microleakage [28]. On the other hand, implants with an internal tapered connection and platform switching have been shown to increase connection fit and stability [28]. However, additional long-term studies are needed to explore the effects of prosthetic loading on peri-implant tissues. Regrettably, the authors were unable to identify any study facilitating a comparative analysis of results concerning the influence of elapsed time on the extent of bone resorption. The scarcity of existing literature addressing this specific aspect underscores the need for further research to elucidate the temporal dynamics of bone resorption in the context of implant-supported prostheses.

In addition to the factors explored in this study, the type of material used for prosthetic restoration also merits attention. Zirconia restorations exhibited higher resorption values in the vertical plane, emphasizing the need for continued investigation into the influence of material characteristics on peri-implant outcomes. While no studies specifically quantified resorption in the mentioned planes, investigations assessing the survival rates of zirconia and metal-ceramic implant supported crowns were identified. Notably, metal-ceramic crowns exhibited a slightly higher survival rate at 5 years compared to their zirconia counterparts [23]. The same systematic review revealed a higher degree of bone resorption associated with zirconia single crowns compared to those made of metal-ceramic, although the observed difference did not reach statistical significance [23]. Based on the current body of knowledge, both zirconia and metal-ceramic materials can be confidently employed for the restoration of dental implants [40]. However, giving the observed differences in resorption patterns between zirconia and metal-ceramic restorations highlight the importance of prosthetic material selection. Clinicians should consider these findings when choosing materials for implant-supported restorations, particularly in cases where preserving peri-implant bone height is a priority. Zirconia restorations may be preferable in situations where minimal vertical resorption is desired, while metal-ceramic restorations could be suitable alternatives if other factors such as esthetics or strength are prioritized.

These findings have practical implications for clinicians involved in implant-supported fixed prosthodontics. The observed differences in resorption patterns between different prosthetic materials and designs underscore the importance of meticulous treatment planning in implant dentistry. Clinicians should carefully consider factors such as patient demographics, aesthetic requirements, occlusal forces, and long-term bone stability when selecting implant components and designing prosthetic reconstructions. Based on the findings, clinicians may prioritize zirconia restorations for patients at higher risk of vertical resorption or select prosthetic designs that minimize cantilever lengths to reduce the risk of peri-implant bone loss over time. The findings also suggest that post-operative care protocols should emphasize meticulous oral hygiene maintenance and regular follow-up evaluations to monitor peri-implant bone health over time. Patients with higher risk factors, such as older age or specific prosthetic designs, may require more frequent follow-up visits to detect and address early signs of peri-implant complications promptly. Additionally, patient education regarding proper oral hygiene practices and the importance of regular dental visits is crucial to minimize the risk of peri-implant complications and optimize long-term implant success. Clinicians should integrate these findings into evidence-based treatment protocols and continuously reassess and adapt treatment strategies based on evolving patient needs and scientific advancements. Additionally, fostering interdisciplinary collaboration among dental professionals, including prosthodontists, periodontists, and oral surgeons, can enhance treatment outcomes and facilitate comprehensive care delivery in implant dentistry.

Acknowledging the limitations of this study, including its retrospective design and focus on a specific patient population, future research endeavors should encompass larger and more diverse cohorts to enhance the generalizability of findings. Longitudinal studies could provide a more comprehensive understanding of the temporal aspects of peri-implant resorption, shedding light on dynamic changes over extended periods.

However, it is the authors’ opinion that this research contributes to the evolving landscape of implant-supported fixed prosthetics by elucidating the multifaceted factors influencing peri-implant resorption. 

## 5. Conclusions

These findings shed light on the intricate interplay between prosthetic material, design, patient age, and gender, and their impact on peri-implant bone health. Specifically, distinct resorption patterns associated with different prosthetic materials and designs were observed, highlighting the importance of personalized treatment planning in optimizing long-term implant success. The implications of our research for clinical practice are profound. Clinicians can use these findings to tailor treatment approaches, select appropriate prosthetic materials and designs, and optimize post-operative care protocols to minimize the risk of peri-implant complications and enhance implant longevity. By integrating evidence-based strategies informed by our research, clinicians can improve patient outcomes and satisfaction in implant dentistry. Moving forward, we call for continued research efforts to further elucidate the underlying mechanisms driving peri-implant resorption dynamics and to explore novel interventions aimed at preserving peri-implant bone health. Additionally, we urge clinicians to embrace a patient-centered approach to implant treatment, incorporating personalized risk assessments and comprehensive care delivery to maximize the success and longevity of dental implants. It is important to underscore the fact that systemic diseases may influence peri-implant outcomes, but our study did not systematically assess their impact. Future research should incorporate detailed patient medical histories and consider systemic diseases as potential confounding variables in analyzing implant outcomes.

## Figures and Tables

**Table 1:** Comparison of resorption and material used for restoration.

Material	Mean ± SD	Median (IQR)	Medium Rank	*p* *
**Mesial-Vertical**				
Zirconia (*p* = 0.007 **)	1.83 ± 1.42	1.65 (0.93–2.43)	86.65	0.355
Metal-ceramic (*p* < 0.001 **)	1.62 ± 1.49	1.36 (0.24–2.35)	78.65	
**Distal-Vertical**				
Zirconia (*p* = 0.466 **)	1.74 ± 1.03	1.76 (1.01–2.57)	85.46	0.456
Metal-ceramic (*p* < 0.001 **)	1.73 ± 1.51	1.65 (0.54–2.47)	79.01	
**Mesial-Horizontal**				
Zirconia (*p* = 0.002 **)	0.64 ± 0.62	0.56 (0–1.10)	82.22	0.794
Metal-ceramic (*p* < 0.001 **)	0.65 ± 0.77	0.54 (0–0.91)	79.98	
**Distal-Horizontal**				
Zirconia (*p* < 0.001 **)	0.61 ± 0.63	0.51 (0–1.07)	81.05	0.932
Metal-ceramic (*p* < 0.001 **)	0.62 ± 0.69	0.49 (0–1.01)	80.33	

SD—Standard deviation, IQR—interquartile range, * Mann-Whitney U Test, ** Shapiro-Wilk Test, significance level at *p* < 0.05.

**Table 3:** Comparison of resorption and type of restoration.

Restoration	Mean ± SD	Median (IQR)	Medium Rank	*p* *
**Mesial-Vertical**				
Single crown (*p* < 0.001 **)	1.59 ± 1.48	1.58 (0.24–2.31)	77.87	0.217
Connected crowns (*p* < 0.001 **)	1.53 ± 1.43	1.22 (0–2.34)	75.88	
Bridge (*p* = 0.025 **)	1.98 ± 1.53	1.86 (0.74–2.79)	91.24	
**Distal-Vertical**				
Single crown (*p* < 0.001 **)	1.81 ± 1.44	1.74 (0.99–2.47)	83.44	0.026
Connected crowns (*p* < 0.001 **)	1.43 ± 1.28	1.39 (0–1.99)	69.45	
Bridge (*p* = 0.029 **)	2.10 ± 1.51	1.90 (0.80–3.03)	93.82	
**Mesial-Horizontal**				
Single crown (*p* < 0.001 **)	0.56 ± 0.56	0.5 (0.5–0.95)	76.90	0.055
Connected crowns (*p* < 0.001 **)	0.55 ± 0.64	0.53 (0–0.94)	74.20	
Bridge (*p* < 0.001 **)	0.93 ± 1.00	0.68 (0.42–1.10)	95.16	
**Distal-Horizontal**				
Single crown (*p* < 0.001 **)	0.68 ± 0.68	0.56 (0–1.19)	85.33	0.283
Connected crowns (*p* < 0.001 **)	0.54 ± 0.69	0.31 (0–1.02)	73.55	
Bridge (*p* < 0.001 **)	0.65 ± 0.66	0.39 (0.22–0.87)	84.88	

SD—Standard deviation, IQR—interquartile range, * Mann-Whitney U Test, ** Shapiro-Wilk Test, significance level at *p* < 0.05.

**Table 4:** Mesial/distal—horizontal/vertical resorption for single crowns, connected crowns, dental bridges.

	Resorbtion	Mean ± SD	Median (IQR)	*p* *
**Single**	Mesial-Vertical (*p* < 0.001 **)	1.59 ± 1.48	1.58 (0.24–2.31)	<0.001
	Distal-Vertical (*p* < 0.001 **)	1.81 ± 1.44	1.74 (0.99–2.47)	
	Mesial-Horizontal (*p* < 0.001 **)	0.56 ± 0.56	0.50 (0–0.95)	
	Distal-Horizontal (*p* < 0.001 **)	0.68 ± 0.68	0.56 (0–1.19)	
**Connected**	Mesial-Vertical (*p* < 0.001 **)	1.53 ± 1.43	1.22 (0–2.34)	<0.001
	Distal-Vertical (*p* < 0.001 **)	1.43 ± 1.28	1.39 (0–1.99)	
	Mesial-Horizontal (*p* < 0.001 **)	0.55 ± 0.64	0.53 (0–0.94)	
	Distal-Horizontal (*p* < 0.001 **)	0.54 ± 0.69	0.31 (0–1.02)	
**Bridge**	Mezial-Vertical (*p* = 0.025 **)	1.98 ± 1.53	1.86 (0.74–2.79)	<0.001
	Distal-Vertical (*p* = 0.029 **)	2.10 ± 1.51	1.90 (0.80–3.03)	
	Mezial-Orizontal (*p* < 0.001 **)	0.93 ± 1.00	0.68 (0.42–1.10)	
	Distal-Orizontal (*p* < 0.001 **)	0.65 ± 0.66	0.39 (0.22–0.87)	

SD—Standard deviation, IQR—interquartile range, * Related-Samples Friedman’s Two-Way Analysis of Variance by Ranks, ** Shapiro-Wilk Test, significance level at *p* < 0.05.

## Data Availability

The data presented in the study are available on request from the corresponding authors. The data are not publicly available due to privacy reasons.
